# Commentary: First-Line Interactive Wound Dressing Update: A Comprehensive Review of the Evidence

**DOI:** 10.3389/fphar.2020.01272

**Published:** 2020-09-09

**Authors:** Linda L. Benskin

**Affiliations:** ^1^ Independent Research for Wound Care in Developing Countries, Austin, TX, United States; ^2^ Ferris Mfg. Corp., Fort Worth, TX, United States

**Keywords:** PolyMem, hydroactive dressings, interactive dressings, polymeric membrane dressings, PMDs, moisture

## Introduction

Although Weller et al., in First-Line Interactive Wound Dressing Update: A Comprehensive Review of the Evidence (Weller et al., 2020), served the wound management community well by ably communicating the importance of the interactive nature of some wound dressings, they misidentified PolyMem*^®^* as a hydroactive dressing. PolyMem dressings are interactive dressings which share some similarities with hydroactive dressings. However, because PolyMem is multifunctional, wound management experts familiar with PolyMem (generic name: polymeric membrane dressings, abbreviated PMDs) discuss these dressings as a distinct dressing type (Mulder, 2009; Thomas, 2010; Dabiri et al., 2014; Denyer et al., 2017; Benskin, 2018). The online resource Weller et al. cite as their primary source of wound dressing information is no exception (Products | Wound Care Handbook). In this resource, PolyMem is described under the “polymeric membrane dressings” section thusly: “A hydrophilic polyurethane matrix with a mild, non-toxic wound cleanser, a soothing moisturizer, a superabsorbent and a semi-permeable film backing. Designed to facilitate healing, relieve pain and reduce inflammation.” (Polymeric membrane dressing | Wound Care Handbook). PolyMem’s components are all completely integrated into the hydrophilic polyurethane matrix to permit even and consistent functionality (Benskin, 2016).

## Attributes of Hydroactive Dressings

Weller et al. found that the additional benefits of hydroactive dressings (balancing moisture) justify placing them in a distinct dressing “form” (subcategory), citing “hydrocolloid and hydroactive wound dressings” (de Vries, 2018). Hydroactive dressings are described as suitable for highly exudating wounds, but not for dry, lightly exudating, or infected wounds, and their ability to expand and contract to accommodate movement is especially helpful for joint wounds (de Vries, 2018). Unlike hydrocolloids, which combine with exudate to form a gel at the wound surface, hydroactive dressings trap exudate in an internal “hydrogel net” while maintaining an appropriately moist wound-dressing interface (de Vries, 2018).

## Similarities Between Hydroactive and Polymeric Membrane Dressings

The cited study does not mention PolyMem or PMDs (de Vries, 2018). However, recognition that PolyMem also traps excess exudate in the dressings while maintaining an optimally moist wound environment may have led Weller et al. to include PolyMem as a hydroactive dressing (Benskin, 2016). Also, like hydroactive dressings, PolyMem is known for being quite flexible, accommodating comfortable joint movement (Benskin, 2015).

## How Multifunctional Polymeric Membrane Dressings Differ From Hydroactive Dressings

PolyMem’s powerful moisture-balancing system includes a super-absorbent locked in the dressings, somewhat similar to hydroactive dressings’ “hydrogel net”(Benskin, 2016). However, PolyMem also works synergistically with the body, slowly releasing glycerol, which moisturizes wound surfaces and pulls fluid from the body into the wound bed, and from there, into the dressing (Benskin, 2016). This water flux allows the moisture balancing system of PMDs to be more powerful than that of hydroactive dressings, in that it can moisturize dry areas of wounds, including easily desiccated structures like tendon or bone, while simultaneously absorbing excess exudate (Benskin, 2016; Benskin, 2018). Excess moisture is redistributed within the hydrophilic polyurethane substrate, and the “intelligent” backing adjusts the water vapor transmission rate based upon the dressing’s saturation (Benskin, 2018).

This water flux is also integral to the interactive wound cleansing system that allows PolyMem to be indicated for all wound types at all stages of healing, including infected wounds—another benefit not shared by hydroactive dressings (Benskin, 2016). Moisture stimulates PolyMem dressings to release a nontoxic surfactant, which breaks the bonds between wound contaminants and the wound surface (Benskin, 2016). The dislodged contaminants are floated into the dressing with the excess wound fluid recruited by the glycerol (Benskin, 2016). This atraumatic continuous cleansing system is so powerful that routine rinsing is not recommended when PolyMem dressings are used, and it allows PolyMem to be recommended for use on infected wounds (Benskin, 2016).

Patients using PolyMem also benefit from its pain relieving properties, which are directly related to PolyMem’s influence upon the nociceptor response, even over intact skin (Cutting et al., 2015). The ability of PolyMem to focus and control inflammation makes it particularly useful for closed tissue injuries, including stage I and Deep Tissue Pressure Injuries (Benskin, 2018).

## Evidence Supporting Polymeric Membrane Dressings as a Unique Dressing Type

The review by Weller et al. concluded that the lack of high-quality evidence for interactive dressings demonstrates a need for well-designed trials (Weller et al., 2020). In contrast, while more trials would certainly be welcomed, over the past 30 years, a large body of evidence has been built to support the use of PolyMem (Benskin, 2018). PolyMem’s unique benefits are discussed in no less than 80 peer-reviewed journal articles and formal consensus documents, including eight true randomized controlled trials, nine less rigorous clinical studies and 23 case studies or case series. Polymeric membrane dressings are recognized as a unique dressing type by the US Agency for Healthcare Research and Quality (Saha et al., 2013).

Weller et al. did not mention a relevant PubMed-indexed review article summarizing the evidence related to polymeric membrane dressings for pressure injuries (Benskin, 2018). They did, however, include Dabiri et al., who join a growing body of wound experts in placing polymeric membrane dressings (PMDs) in a unique subcategory because of PolyMem’s extensive range of functions. (Lee, 2005; Mulder, 2009; Thomas, 2010; Saha et al., 2013; Dabiri et al., 2014; Denyer et al., 2017; Hess, 2019; Croitoru et al., 2020). In addition, the 2019 update of the Prevention and Treatment of Pressure Ulcers/Injuries Guidelines recommends PMDs, recognizing them as a unique dressing type (European Pressure Ulcer Advisory Panel et al., 2019).

## Conclusion

While PolyMem does share the function of moisture balancing with hydroactive dressings, there are many significant differences (Benskin, 2018), The benefits patients experience when using polymeric membrane dressings, which are well beyond those experienced with hydroactive dressings, justify yet another distinct dressing subcategory. In addition to the benefits listed by Weller et al. for hydroactive dressings, polymeric membrane dressings can be used on infected wounds, and have the demonstrated ability to continuously cleanse wounds, moisturize dry wounds, focus the inflammation at the site of injury, and relieve pain by interacting with the nociceptor system (Benskin, 2015; Benskin, 2016). These benefits are substantial, and unlike those of hydroactive dressings, they are backed by a strong evidence base, including randomized controlled trials (Benskin, 2016).

## Author's Note

The author discovered PolyMem® among the donated dressings while working for 5 years in a remote clinic in northern Ghana, West Africa. As a result of her extensive experience using PolyMem on well over one thousand patients, LB became so passionate about the benefits of these unique dressings that she is currently an employee of Ferris Mfg. Corp., makers of PolyMem dressings.

## Author Contributions

The author confirms being the sole contributor of this work and has approved it for publication.

## Conflict of Interest

LB is currently an employee of Ferris Mfg. Corp., makers of PolyMem dressings.
